# Special Report on Electrical Standards: Report on the 17^th^ Session Of the Consultative Committee On Electricity

**DOI:** 10.6028/jres.092.005

**Published:** 1987-02-01

**Authors:** B. N. Taylor

**Affiliations:** National Bureau of Standards Gaithersburg, MD 20899

**Keywords:** Consultative Committee on Electricity, electrical units, Josephson effect, ohm, quantum Hall effect, volt

## Abstract

This report provides the background for and summarizes the main results of the 17th session of the Consultative Committee on Electricity (CCE) of the International Committee of Weights and Measures held in September 1986. Included are decisions made by the CCE which promise to have a profound effect on the standardization of national representations of the volt and ohm and thus on the international compatibility of electrical measurements. In particular, on January 1, 1990, worldwide changes in the basis for such representations are planned which will lead to an increase in the U.S. legal unit of voltage of about 9 parts-per-million (ppm) and in the U.S. legal unit of resistance of about 1.5 ppm.

## 1. Background

The 17th session of the Consultative Committee on Electricity (CCE) of the International Committee of Weights and Measures (CIPM) met September 16–18, 1986, at the International Bureau of Weights and Measures (BIPM), which is located in Sèvres (a suburb of Paris), France. NBS Director Ernest Ambler, who was recently elected President of the CCE by the CIPM, chaired the meeting and the author attended as NBS representative.

**Figure 1:**
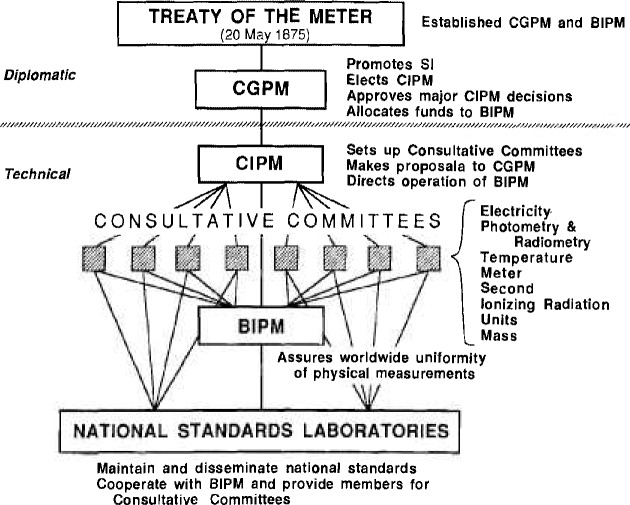
Schematic depiction of how basic measurement units and standards are coordinated internationally. (Treaty of the Meten *La Convention du Mètre;* CGPM: *Conférence Général des Poids et Mesures* or General Conference of Weights and Measures; CIPM: *Comité International des Poids et Mesures* or International Committee of Weights and Measures; BIPM: *Bureau International des Poids et Mesures* or International Bureau of Weights and Measures.)

The CCE is one of eight CIPM Consultative Committees which together cover most of the areas of basic metrology [1]^1^ (see fig. 1). These committees, which may form temporary or permanent ‘Working Groups’ to study special subjects, coordinate the international work carried out in their respective fields, assist the CIPM in supervising the work of the BIPM in these fields, and propose recommendations concerning amendments to be made to the definitions and values of units. The CIPM acts directly on these recommendations or submits them for approval to the General Conference of Weights and Measures (CGPM) if they will have a broad impact.

The BIPM, whose principal task is to ensure worldwide uniformity of physical measurements, was established by the Treaty of the Meter signedin Paris on May 20, 1875. (The number of signatories, originally 17, has now grown to almost 50.) The BIPM is supervised by the CIPM which in turn is under the authority of the CGPM. The CGPM consists of delegates from all of the member countries of the Treaty of the Meter and presently meets every four years. The CIPM consists of 18 members each representing a different country and presently meets every year. In general, a consultative committee meets every few years, has as its president a member of the CIPM, and is composed of delegates from the major national standards laboratories as well as from specialized institutes, and individual members appointed by the CIPM.

**Figure 2:**
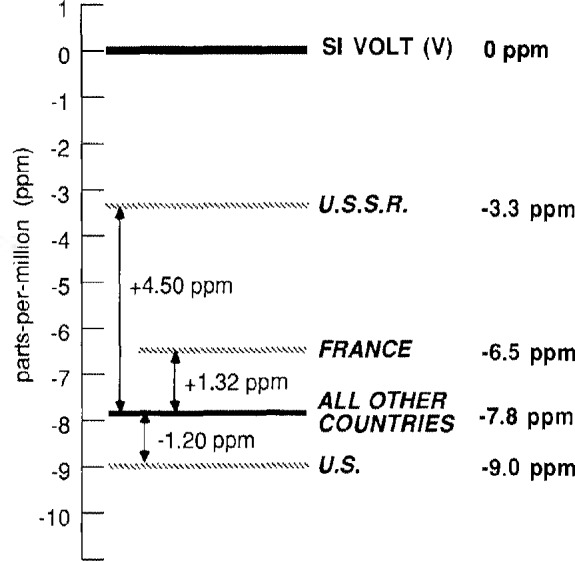
Graphical comparison of the representation of the SI volt of various countries based on the Josephson effect, V_LAB_, and of V_LAB_ with the SI volt, V. The value of V_LAB_ indicated by ‘All Other Countries’ is based on the value of the Joseph-son frequency-voltage ratio *2e/h* recommended by the CCE of the CIPM in 1972: (2*e*/*h*)_CCE–72_=483594.0 GHz/V. (See footnote 2 for those countries which use this value.)

The focus of the 17th session of the CCE, which was attended by some 30 individuals from 15 countries, was the use of the Josephson and quantum Hall effects to define and maintain national or laboratory representations of the units of electrical potential difference and resistance of *Le Système International d’Unités* or International System of Units (abbreviated SI): the SI volt (V) and SI ohm (Ω), respectively [2]. (The national representations are usually designated by the symbol V_LAB_ and Ω_LAB_ where LAB stands for the acronym of the national standards laboratory of the country in question, e.g., NBS.) More specifically, at its 16th session in March 1983 the CCE had concluded that [3] (see fig. 2):

(i) The value 483594.0 GHz/V for the Joseph-son frequency-voltage ratio *2e/h (e* is the elementary charge and *h* is the Planck constant) which it had recommended at its 13th session in 1972 [4–7] for defining and maintaining national representations of the SI volt is significantly in error. (Current evidence indicates that (2*e/h*)CCE-72 is about eight parts-per-million or 8 ppm smaller than the SI value of *2e/h* and thus V_LAB_ is about 8 ppm smaller than V for those national laboratories which use the CCE value [2,8].)
(ii) It was highly likely that the recently discovered quantum Hall effect would soon be developed to the point that the quantized Hall resistance R_H_ = *h/e*^2^ = 25812.8 Ω could be used to define and maintain national units of resistance consistent with the SI ohm to within a few tenths of a ppm. (More recent work indicates that a few hundredths of a ppm is quite feasible [9].)

The CCE was also aware of the following:

(iii) Four different values of *2e/h* are in use in the national laboratories [2]. [The U.S., France, and the U.S.S.R. use values of *2e/h* which differ by −1.20 ppm, +1.32 ppm, and +4.50 ppm, respectively from (2*e*/*h*)_CCE-72_ and hence the national voltage units of these countries differ by these amounts from the national units of those countries which use (*2e*/*h*)_CCE-72_.^2^]
(iv) The various national units of resistance, most of which are based on the mean resistance of a group of precision wire-wound resistors, differ from each other and the SI unit by up to several ppm and some are drifting in excess of 0.05 ppm per year. (Current evidence indicates that on January 1, 1986, the various ω_LAB_ were from 0.2 ppm larger to 3.3 ppm smaller than *Ω* and *dΩ*_LAB_/*dt* lies in the range −0.07 to +0.07 ppm/year [8].)

As a consequence of (i) through (iv), the CCE decided at its 16th session in 1983 to hold its 17th session in 1986 in order to consider the possibility of recommending for adoption a new value for *2e/h*, consistent with the SI, to be used by every laboratory which employs the Josephson effect to define and maintain its representation of the SI volt; and a value of *R*_H_, consistent with the SI, to be used by every laboratory which chooses to employ the quantum Hall effect to define and maintain its representation of the SI ohm.^3^

Considerable preparatory work was carried out during the two years prior to the CCE’s 17th session:

- Immediately after the formal close of the 1984 Conference on Precision Electromagnetic Measurements (CPEM 84, held August 20–24, 1984, in Delft, The Netherlands), active research workers from the national standards laboratories and other interested parties met informally to discuss values of 2*e*/*h* and *R*_H_. Many new and relevant results were also presented at the conference itself [10].
- In the U.S., NBS Director Ambler sent a letter in early 1985 to over 30 U.S. organizations, companies, and individuals representing industry, government, science, and academia and having an interest in basic electrical measurements and standards at the highest levels of accuracy. The purpose of the letter was to give the U.S. scientific and technological communities the opportunity, well in advance of the 1986 CCE meeting, to provide NBS with advice and guidance on the subject of changing the U.S. electrical units. (The U.S. Legal Volt V_NBS_ would increase by about 9 ppm and the U.S. Legal Ohm ω_NBS_ by about 1.5 ppm if the CCE were to recommend a new value for *2e/h* consistent with the SI and a value of *R*_H_ also consistent with the SI.) The comments received were presented during CPEM 86 (held at NBS Gaithersburg, June 23–27, 1986) at a special session with active audience participation entitled “Changes in the Electrical Units.” In addition to the U.S. presenter, viewpoints from other countries were given by speakers from the Federal Republic of Germany, Japan, and the U.K.^4^ The three key points which emerged from this session were:
  - Changing national voltage and resistance units will outweigh the considerable costs of making the changes if and only if complete international uniformity of all the national units of the industrialized countries is achieved.
  - The changes must be well justified by the data; all of the available information must be analyzed by knowledgeable experts and no changes made unless the uncertainties in the values of *2e/h* and *R*_H_ are sufficiently small that it is highly unlikely that further changes will be necessary in the forseeable future.
  - At least one year should be allowed from the date of the official announcement that the changes will take place to the date of their actual implementation so that industry will have sufficient time to prepare itself properly.
- Many researchers took the occasion of CPEM 86 to present their latest results on values of *2e/h* and *R*_H_ [11].
- There was an informal meeting of active research workers and other interested parties to discuss values of *2e/h* and *R*_H_ just after the close of CPEM 86 as there was at the close of CPEM 84.
- Fifty-nine documents were submitted to the CCE by the CCE members (i.e., by the national standards laboratories of the member countries) in support of the CCE’s deliberations. These documents included new results as well as comments on the key issues facing the CCE concerning the adoption of a new value for *2e/h* and a value of *R_H_.*
- A detailed provisionary agenda containing some 25 items was prepared for the 1986 CCE meeting with extensive supporting material in succinct, summary form [12].

## 2. CCE 17th Session Discussions and Principal Decisions

### 2.1 Josephson Effect

The principal Josephson effect topics reviewed in detail by the CCE were [12] (i) the value of *2e*/*h* used by each national standards laboratory to maintain its representation of the SI volt and the accuracy achieved; (ii) the observed agreement among national voltage standards based on the Josephson effect; (iii) the uncertainties associated with intercomparing these voltage standards using transportable standard cells and Zener diode devices; (iv) the values of *2e*/*h* in SI units and their uncertainties obtained by direct force balance measurements and indirectly from fundamental constant determinations; (v) the prospects for future SI values of *2e*/*h* with their expected uncertainties and dates of availability; and (vi) the need for further intercomparisons of national voltage standards and Josephson apparatus.

With regard to (i), the uncertainties achieved were noted to be generally in the range 0.01 to 0.1 ppm^5^, although Josephson arrays may enable uncertainties smaller than 0.01 ppm to be achieved routinely [13]. Under (ii) and (iii), it was concluded that the agreement between national Josephson voltage standards was generally better than 0.1 ppm but that it was difficult to demonstrate this level of consistency using volt transfer standards. With regard to (iv) and (v), the CCE decided that although the data currently available (both direct and indirect) could provide a value of *2e*/*h* in SI units with an uncertainty of about 0.2 ppm, additional data expected to be available within two years would significantly increase confidence in the reliability of the value. Finally, under (vi), the CCE concluded that a formal, broadly based, international comparison of national units of voltage would not be useful because of the unreliability of volt transfer standards but that those laboratories involved in determinations of *2e*/*h* should attempt to compare their units using the best transfer standards at their disposal.

The result of the CCE’s review of these Joseph-son effect topics was the following formal declaration:

#### Declaration E1 (1986)

Concerning the Josephson effect for maintaining the representation of the volt.

The Comité Consultatif d’Electricité

**recognizes**

– that as an organ of the Convention du Mètre one of its responsibilities is to ensure the propagation and improvement of the SI, the unit system in use throughout the world,
– that worldwide uniformity and constancy over a long period of time of national representations of the volt are of great technical and economic importance to commerce and industry,
– that many national standards laboratories use the Josephson effect to maintain a highly stable representation of the volt but that not all use the same value for the quotient frequency to voltage,
– that the value of this quotient (483594.0 GHz/V) declared by the CCE in 1972 and which most national laboratories use to maintain representations of the volt is now known to be in error by a significant amount,
– that various laboratories have carried out direct realizations of the volt or determinations of fundamental constants which can yield an indirect value of *2e/h* in SI units,
– that other national laboratories expect shortly to complete similar realizations or determinations,
  **is of the opinion**
– that the value of the quotient frequency to voltage used to maintain a realization of the volt by means of the Josephson effect must be consistent with the SI,
– that a new value, more consistent with SI, can soon be adopted for use by all laboratories,
– that this new value should be adopted simultaneously by all countries concerned, in consequence, the CCE
– **establishes** a Working Group charged with making a proposal to the CCE for a new value to be based upon all relevant data that become available before June 15, 1988,
– **decides** to meet in September 1988 with a view to recommending the new value of this quotient to come into effect on January 1, 1990,
– **gives notice** that the new value is likely to be higher than the present one by about 8 parts in 10^6^, furthermore, the CCE
– **recommends** that national laboratories vigorously pursue their work on the realizations of the volt, the intercomparison of these realizations, and the determination of the constants in question and communicate without delay all their results to the Working Group,
– **recommends** that laboratories do not change their value for this quotient until the new value comes into effect,
– **believes** that the value to be adopted will be sufficiently accurate, in terms of SI, that no further change will be required in the foreseeable future.

The unsatisfactory state of volt transfer standards led the CCE also to develop Recommendation E 1 (1986), the thrust of which is that the national standards laboratories “actively pursue the study and improvement of transportable standards with which the volt may be transferred from one laboratory to another…” Both Declaration E 1 (1986) and Recommendation E 1 (1986) were subsequently approved by the CIPM at its October 1986 meeting [14].

### 2.2 Quantum Hall Effect

The principal quantum Hall effect (QHE) topics reviewed in detail by the CCE were [12] (i) the values and accuracies achieved in measurements of the quantized Hall resistance *R*_H_ = *h*/*e*^2^ in terms of national representations of the ohm; (ii) the values of *R*_H_ in SI units and their uncertainties obtained by direct calculable capacitor-based measurements and indirectly from fundamental constant determinations; (iii) the prospects for future SI values of *R*_H_ with their expected uncertainties and dates of availability; (iv) the results of recent comparisons of national units of resistance using transportable resistance standards; (v) the agreement among the present values of *R*_H_ in laboratory units and the agreement between various realizations of the SI ohm based on the calculable capacitor; (vi) the precautions required to ensure reliable results from a quantized Hall resistance sample and the availability of good samples; and (vii) the need for further intercomparisons of national units of resistance.

With regard to (i), most laboratories were able to determine *R*_H_ in terms of their national ohm with an uncertainty in the range 0.02 to 0.1 ppm. Under (ii) and (iii), the uncertainties of the values of *R*_H_ in SI units, both direct and indirect, varied between 0.020 to 0.32 ppm, and most values agreed with the value having the smallest uncertainty within 0.2 ppm. A number of new and possibly more accurate results could be expected within two years. With regard to (iv), a 0.05 ppm transfer uncertainty or even better can apparently be achieved if the transport resistors are carefully selected and used. Under (v), the CCE concluded that most measurements of *R*_H_ in laboratory units agreed within an uncertainty of 0.2 ppm but that the agreement among realizations of the SI ohm was somewhat worse. With regard to (vi), the CCE decided that while tests were available which could be used to ensure reliable results from a particular quantized Hall resistance sample, and that a number of good samples are already in hand, increased understanding of the QHE as well as additional metrologically useful samples were highly desirable. Finally, under (vii), the CCE decided that it would be useful to conduct an international comparison of 1-Ω resistance standards to facilitate the comparison of measurements of *R*_H_ in laboratory units.

The result of the CCE’s review of these quantum Hall effect topics was the following formal declaration:

#### Declaration E 2 (1986)

Concerning the quantum Hall effect for maintaining a representation of the ohm.

The Comité Consultatif d’Electricité

**recognizes**

– that as an organ of the Convention du Mètre one of its responsibilities is to ensure the propagation and improvement of the SI, the unit system in use throughout the world,
– that worldwide uniformity and constancy over a long period of time of national representations of the ohm are of great technical and economic importance to commerce and industry,
– that the application of the quantum Hall effect as a means of maintaining a stable representation of the ohm is being developed rapidly in many national standards laboratories,
– that the quantum Hall effect is providing very reproducible results from one laboratory to another, but that the number of usable samples available is insufficient for present needs,
– that experience is leading to tests that provide assurance of both reproducible and accurate results from a selected sample,
– that no laboratory has yet adopted a value of the quantized Hall resistance *R_H_* to maintain its laboratory representation of the ohm,
– that various laboratories have determined *R*_H_ in SI units using both the calculable capacitor and determinations of fundamental constants,
– that additional results for *R*_H_ in SI units are expected to become available in the near future,
  **is of the opinion**
– that the same value of *R*_H_ should be adopted simultaneously by all those laboratories that decide to use the quantized Hall resistance as their representation of the ohm,
– that this value should be consistent with SI,
– that such a value can soon be adopted, in consequence, the CCE
– **establishes** a Working Group charged with making a proposal to the CCE for a value of *R*_H_ to be based upon all relevant data that become available up unitl June 15, 1988 and with developing detailed guidelines for the proper use of the quantum Hall effect to maintain a representation of the ohm,
– **decides** to meet in September 1988 with a view to recommending the value of *R*_H_ to come into effect on January 1, 1990,
– **gives** notice that the adoption of this value for *R*_H_ may lead to a change in national and the BIPM representations of the ohm; this change should in general not exceed 2 parts in 10^6^, furthermore, the CCE
– **recommends** that national laboratories
  – vigorously pursue their work to understand better the quantum Hall effect,
  – encourage the increased availability and distribution of good quantum Hall effect samples,
  – determine the value of *R*_H_ in SI units both by the direct realization of the ohm and the determination of appropriate fundamental constants,
  – carry out bilateral comparisons as seem appropriate, and communicate without delay all their results to the Working Group,
– **recommends** that the BIPM organize during 1987/88 an international comparison of one ohm resistance standards in connection with the quantum-Hall effect work,
– **recommends** that no laboratory should adopt a value of *R*_H_ for its representation of the ohm or use the quantum Hall effect to alter the present drift rate until the recommended value comes into effect,
– **believes** that the value to be recommended in 1988 will be sufficiently accurate, in terms of SI, for no change to be required in the foreseeable future.

The less than satisfactory current state of understanding of the QHE and the limited availability of good samples led the CCE also to develop Recommendation E 2 (1986) which encourages (a) studies of QHE sample manufacture and characterization, (b) the provision of an adequate supply of high quality QHE devices for metrological purposes by industry and research laboratories, (c) better theoretical and experimental understanding of the QHE, and (d) comparisons of QHE devices under the auspices of the BIPM. Both Declaration E 2 (1986) and Recommendation E 2 (1986) were also subsequently approved by the CIPM at its October 1986 meeting [14].

## 3. Conclusion

If all proceeds as planned, that is, if the several new values for *2e*/*h* and *R*_H_ in SI units which are expected to become available by June 15, 1988, agree with earlier results within acceptable limits, then the CCE at its 18th Session in September 1988 will officially recommend for adoption a new value for the Josephson frequency-voltage ratio *2e*/*h* consistent with the SI, and a value of the quantized Hall resistance R_H_ = *h*/*e*^2^ also consistent with the SI, to be used by all national standards laboratories and the BIPM to define and maintain their representations of the volt and ohm. These new values, which would be implemented simultaneously throughout the world starting January 1, 1990, are anticipated to have an uncertainty of between 0.1 and 0.3 ppm. Moreover, the uncertainty associated with using the Josephson and quantum Hall effects to define and maintain representations of the volt and ohm should generally be in the range 0.01 to 0.1 ppm. As a consequence, starting January 1, 1990, the practical electrical units for voltage, resistance, and current of most industrialized countries will be equivalent within an uncertainty no greater than about 0.1 ppm and these units will be consistent with their respective SI units within an uncertainty no greater than about 0.3 ppm. Although implementing these new representations will require adjusting a large industrial inventory of standards and instruments by significant amounts (e.g., in the United States the U.S. Legal Volt will increase about 9 ppm and the U.S. Legal Ohm about 1.5 ppm), the benefits of international uniformity of electrical measurements and their consistency with the SI which will result from the unit changes should completely outweigh the costs of making them.

## Footnotes

1 Numbers in brackets indicate literature references.

2 These include Australia, Canada, the Federal Republic of Germany, Finland, the German Democratic Republic, Italy, Japan, The Netherlands, and the U.K., as well as the BIPM.

3 Basing *Ω*_LAB_ on *R*_H_ would, of course, eliminate the drift in ω_LAB_ (i.e., make *dΩ*_LAB_*/dt* = 0).

4 Written versions of the talks given during the session will appear in Ref. [11].

5 Throughout this paper, all uncertainties are one standard deviation estimates.

## References

[1] (1983). Com. Intl. Poids Mes. Com. Consult d’Electricité. Trav 16º Session, 1983.

[2] Taylor BN (1986). J Res Natl Bur Stand.

[3] Giacomo P (1984). Metrologia.

[4] (1972). Com. Intl. Poids. Mes. Com. Consult. d’Electricité. Trav 13º Session, 1972.

[5] (1972). P. V. Séances Com. Intl. Poids Mes. 61º Session.

[6] (1975). Com. Intl. Poids Mes. Com. Consult. d’Electricité. Trav 14º Session, 1975.

[7] (1975). P. V. Séances Com. Intl. Poids Mes. 64º Session.

[8] Taylor BN, Witt TJ (1986). Document CCE/86–46 submitted to the 16º Session of th. Comité Consultatif d’Electricité. CIPM.

[9] Delahaye F, Dominguez D, Alexandre F, Andre JP, Hirtz JP, Razeghi M (1986). Metrologia.

[b10-jresv92n1p55_a1b] 10See the relevant papers in IEEE Trans. Instrum Meas. **IM–34** (June 1985).

[11] (1987). IEEE Trans Instrum Meas.

[12] Com. Intl. Poids Mes. Com. Consult. d’Electricité. Trav 17º Session, 1986.

[13] Hamilton CA, Kautz RL, Steiner RL, Lloyd FL (1985). IEEE Device Lett.

[14] (1986). P. V. Séances Com. Intl. Poids Mes. 75º Session.

